# Defining benchmarks for robotic-assisted low anterior rectum resection in low-morbid patients: a multicenter analysis

**DOI:** 10.1007/s00384-021-03988-6

**Published:** 2021-07-09

**Authors:** Jan-Hendrik Egberts, Jan-Niclas Kersebaum, Benno Mann, Heiko Aselmann, Markus Hirschburger, Julia Graß, Thomas Becker, Jakob Izbicki, Daniel Perez

**Affiliations:** 1 Clinic for Visceral Surgery, Israelitisches Krankenhaus Hamburg, Hamburg, Germany; 2 Clinic for General, Visceral, Thoracic, Transplantation, and Pediatric Surgery, University Hospital Schleswig–Holstein, Campus Kie, Kiel, Germany; 3 Clinic for Visceral Surgery, Augusta-Kranken-Anstalten Bochum, Bochum, Germany; 4 Clinic for General, Visceral, and Vascular Surgery, KRH Klinikum Robert Koch Gehrden, Gehrden, Germany; 5 Clinic for General, Visceral, and Thoracic Surgery, Clinic Worms, Worms, Germany; 6 Clinic for General, Visceral, and Thoracic Surgery, University Hospital Hamburg-Eppendorf, Hamburg, Germany

**Keywords:** Benchmarking, Rectal cancer, Rectum resection, RLAR, Robotic low anterior rectum resection, Robotic surgery

## Abstract

**Purpose:**

To define the best possible outcomes for robotic-assisted low anterior rectum resection (RLAR) using total mesorectal excision (TME) in low-morbid patients, performed by expert robotic surgeons in German robotic centers. The benchmark values were derived from these results.

**Methods:**

The data was retrospectively collected from five German expert centers. After patient exclusion (prior surgery, extended surgery, no prior anastomosis, hand-sewn anastomosis), the benchmark cohort was defined (n = 226). The median with interquartile range was first calculated for the individual centers. The 75th percentile of the median results was defined as the benchmark cutoff and represents the “perfect” achievable outcome. This applied to all benchmark values apart from lymph node yield, where the cutoff was defined as the 25th percentile (more lymph nodes are better).

**Results:**

The benchmark values for conversion and intraoperative complication rates were ≤ 4.0% and ≤ 1.4%, respectively. For postoperative complications, the benchmark was ≤ 28% for “any” and ≤ 18.0% for major complications. The R0 and complete TME rate benchmarks were both 100%, with a lymph node yield of > 18. The benchmark for rate of anastomotic insufficiency was < 12.5% and 90-day mortality was 0%. Readmission rates should not exceed 4%.

**Conclusion:**

This outcome analysis of patients with low comorbidity undergoing RLAR may serve as a reference to evaluate surgical performance in robotic rectum resection.

## Introduction

Rectal resection, in addition to emerging total neoadjuvant therapy [1], is currently the common curative therapy for localized rectal carcinoma [2]. Robotic-assisted low anterior rectum resection (RLAR) can overcome many known limitations of conventional laparoscopy (LLAR). The feasibility and safety of RLAR are now well established, and there is growing evidence that it may offer better peri- and postoperative outcomes compared to LLAR [3]. A meta-analysis published by Han et al. in 2020, which compared the perioperative outcomes of LLAR and RLAR from eight RCTs involving 999 patients, showed that while RLAR led to significantly longer operative time, the conversion rate was lower [4]. However, most of the available literature consists of retrospectively collected datasets, including patients who are operated within the surgeon’s learning curve for RLAR to increase the cohort. Thus, results often demonstrate longer operative times, increased peri- and postoperative complications, and at times worse oncologic outcomes. The only prospective randomized controlled trial (RCT) to compare conversion rates between RLAR and LLAR (the ROLARR study) also had this weakness; participating surgeons were only required to have performed 25 robot-assisted procedures [5]. Thus, it can be assumed that the incomplete learning curve had a negative impact on the surgical results. Furthermore, the implementation of an RCT is difficult. Centers are specialized, so that a comparison between LLAR and RLAR within one center is rarely possible. In addition, there has been an increase in the number of patients actively deciding the surgical technique; thus, generation of two equivalent study arms is often problematic.

To enable a well-founded evaluation of a new technique, standardization is required after implementation. This enables efficient training and further education of the entire surgical team, but also requires regular re-evaluation and further development. For robot-assisted colorectal surgery, this is done at regular intervals by internal reviews of five German centers in which all surgeons work as proctors for Intuitive and therefore have proven expertise in robot-assisted colorectal surgery. All centers operate according to a standardized refined surgical technique [6], which is a full robotic approach without laparoscopic assistance. Our study aims to evaluate the perioperative outcomes of RLAR after completion of the learning curve in an ideal cohort of patients, and thus establish the first benchmark values worldwide that can be used as a comparison for other centers or even other techniques.

## Methods

### Data collection

Data were collected from the five German proctor centers (University Hospital Schleswig–Holstein, Campus Kiel, University Hospital Eppendorf, KRH Klinikum Robert Koch Gehrden, Augusta-Kranken-Anstalten Bochum, and Klinikum Worms; Table 1). To map the learning curve overcomes, all patients were included after the first 100 robot-assisted procedures performed by each surgeon. Therefore, patients operated by other surgeons in the centers, who were within their learning curve, were not included. The centers each contributed the outcome of one experienced surgeon, except center two, where two surgeons performed the surgeries. Data entry into a standardized questionnaire was performed by the centers. The data collected consisted of patient demographics, operative date, operative time, technical characteristics, peri-, postoperative, and oncologic outcomes, conversion rates, readmission, and 30- and 90-day mortalities. If available, additional data were entered for follow-up. These were then analyzed anonymously at Center 1. A positive ethical vote was available for all participating hospitals.

**Table 1:** Participating centers

University Hospital Schleswig–Holstein, Campus Kiel	Clinic for General, Visceral, Thoracic, Transplantation, and Pediatric Surgery	Prof. Dr. med. Jan-Hendrik Egberts
University Hospital Eppendorf	Clinic for General, Visceral, and Thoracic Surgery	Prof. Dr. med. Daniel Perez
KRH Klinikum Robert Koch Gehrden	Clinic for General, Visceral, and Vascular Surgery	Dr. Heiko Aselmann
Augusta-Kranken-Anstalten Bochum	Clinic for Visceral Surgery	PD Dr. med. Benno Mann
Clinic Worms	Clinic for General, Visceral, and Thoracic Surgery	PD Dr. Markus Hirschburger

### Study cohort and inclusion criteria for low comorbidity

**Fig. 1:**
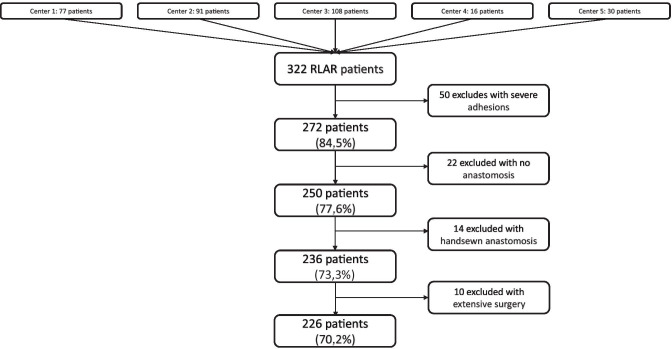
Number of patients included per center and exclusion criteria. RLAR, robot-assisted low anterior rectum resection

The overall cohort consisted of 322 patients from five centers who underwent surgery between January 2013 and January 2020. The median age was 64 (interquartile range (IQR) 56–73) years, and the median body mass index (BMI) was 25.9 (IQR 22.6–28.6) kg/m^2^. The proportion of men was 59.9% (n = 193). To define the benchmark cohort, patients who had prior surgery were excluded (50 patients). Further exclusion criteria were primary discontinuity resection (22 patients), non-machine anastomosis (14 patients), and procedure extension (10 patients; atypical liver resection (n = 2), atypical lung resection via video-assisted thoracoscopic surgery (n = 1), uterine myoma resection (n = 1), multivisceral resection and hysterectomy (n = 1), bladder resection (n = 1), seminal vesicle resection (n = 1), Meckel’s diverticulum resection (n = 1), peritonectomy (n = 1), and creation of a colonic pouch-anal anastomosis (n = 1)). The inclusion criteria are listed in Fig. 1.

### Performance metrics for benchmarking

Primary endpoints for the benchmark analysis were intraoperative complications and conversion rates, positive circumferential resection margin (CRM), total mesorectal excision (TME) quality, and lymph node yield. Pathologic examination was performed according to the guidelines of the German Cancer Society (DKG) [2], which are based on international standards. The TME quality was assessed by an independent pathologist using a standardized procedure that was checked and certified externally.

Secondary endpoints consisted of postoperative Clavien-Dindo complications (CDC), split by “any” complication as well as major complications (CDC ≥ III), the anastomosis insufficiency rate, readmission within 90 days, and the 30- and 90-day mortalities.

### Statistical analysis

Data are presented as numbers (n) with proportions (%) or as median and IQR for continuous variables. For subgroup analysis, the chi-squared test or T-test was used where appropriate. Survival rates were calculated using the Kaplan–Meier function. All p-values were two-sided and considered significant at *p* ≤ 0.05.

Benchmark values were calculated solely from the benchmark cohort (n = 226). We first calculated the median with IQR for the individual centers. From those median, the 75th percentile was found and those were defined as our benchmark values. Thus, outcome parameters above the benchmark value (75th percentile) indicate high morbidity, whereas outcome parameters below the benchmark value indicate acceptable morbidity. This was applied to all benchmark values apart from the lymph node yield, where the cutoff was defined as the 25th percentile, because a higher lymph node yield is better.

We also conducted descriptive statistics for peri-and postoperative parameters where applicable.

Statistical analysis was performed using Statistical Package for Social Sciences software (version 26.0, SPSS Inc., Chicago, IL).

## Results

### Basic characteristics of benchmark patients

The benchmark group consisted of 226 patients; 133 patients (58.9%) were men, and the median age was 64 (IQR 49–70) years with a median BMI of 25.8 (IQR 22.7–28.2) kg/m^2^. The remaining patient characteristics are outlined in Table 2.

**Table 2:** Patient characteristics

	Benchmark patients (n = 226)	Excluded patients (n = 96)	p value
Age (years), median (IQR)	64 (49–70)	66 (60–75)	n.s
BMI (kg/m^2^), median (IQR)	25.8 (22.7–28.2)	26.0 (22.0–29.3)	n.s
Male, n (%)	133 (58.9)	53 (55.2)	n.s
ASA status, n (%)			0.022
Grade I	12 (5.3)	2 (2.1)	
Grade II	140 (61.9)	47 (49.0)	
Grade III	70 (31.0)	45 (46.9)	
Grade IV	4 (1.8)	2 (2.1)	
Histology, n (%)			n.s
Adenosquamous carcinoma	220 (97.3)	91 (94.8)	
Other type of malignancy	4 (1.8)	4 (4.2)	
Benign	2 (0.9)	1 (1.4)	
Tumor size (mm), median (IQR)	35.2 (20.0–40.0)	29.5 (15.0–40.0)	n.s
Missing data, n (%)	42 (18.6)	14 (14.6)	
Preoperative therapy, n (%)			0.001
Radiochemotherapy	69 (30.5)	49 (51.0)	
UICC Stages, n (%)			n.s
0	15 (6.6)	9(9.4)	
I	76 (33.6)	29 (30.2)	
IIA	50 (22.1)	14 (14.6)	
IIB	1 (0.4)	3 (3.1)	
IIIA	17 (7.5)	7 (7.3)	
IIIB	31 (13.7)	11 (11.5)	
IIIC	18 (8.0)	7 (7.3)	
IV	18 (8.0)	16 (16.7)	

*IQR* interquartile range, *n.s.* not significant

The indication for rectal resection was carcinoma in 224 patients (99.1%), with adenocarcinoma being the most common tumor entity (97.3%). Only 69 patients (30.5%) received neoadjuvant therapy prior to resection. The remaining 157 patients underwent primary surgery.

### Intraoperative outcomes in benchmark patients

The median operative time was 266 (IQR 211–310) min (Table 3). In 75 patients (33.2%), a so-called dual-docking procedure with intraoperative repositioning and redocking was performed. One patient experienced intraoperative bleeding (requiring conversion to open rectal resection) and another patient experienced an unspecified intraoperative complication. Conversion was performed in eight cases (4.5%), although only one was tumor-associated (UICC II, R0, CRM negative); the others were converted because of adhesions (n = 3), incidental findings of infrarenal aortic aneurysm (n = 1), unclear anatomy (n = 1), anastomosis creation (n = 1), and suturing over an insufficient anastomosis (n = 1). Two of these cases were converted to laparoscopy (for suturing the anastomosis and in the case of the aortic aneurysm) and the remaining six to laparotomy.

**Table 3:** Intraoperative outcomes

	Benchmark patients (n = 226)	Excluded patients (n = 96)	p value
Dual docking, n (%)	75 (33.2)	29 (30.2)	n.s
Duration of surgery (min), median (IQR)	266 (211–310)	276 (215–328)	n.s
Intraoperative complications, n (%)	2 (0.9)	5 (5.2)	0.017
Bleeding	1 (0.5)	0	
Not specified	1 (0.5)	4 (4.2)	
Conversions, n (%)	8 (4.5)	10 (10.4)	0.015
Tumor associated	1 (0.4)	5 (5.2)	
Not tumor associated	7 (3.1)	5 (5.2)	
Conversion to laparoscopy	2 (0.9)	1 (1.4)	
Conversion to laparotomy	6 (2.7)	9 (9.4)	
Distance of anastomosis from anal verge (cm), median (IQR)	5.8 (4.0–7.0)	4.8 (3–6)	n.s
Missing data, n (%)	110 (48.7)	57 (59.4)	
Primary ileostomy, n (%)	163 (72.1)	77 (80.2)	n.s

*IQR* interquartile range, *n.s.* not significant

The mean anastomosis height was 5.8 (IQR 4.0–7.0) cm from the ano cutaneous line, but data were missing in 110 patients (48.7%). A protective ileostomy was created in 163 cases (72.1%).

### Postoperative outcomes in benchmark patients

Overall morbidity at 30 days was 29.2% (n = 66), of which 14.2% (n = 32) suffered a major complication (CDC ≥ III). The readmission rate within 90 days of discharge was 4% (n = 9), two of which were non-surgery associated (one patient for planned liver metastasectomy and the other with symptomatic ascites due to tumor progression). Five patients showed late insufficiency, which was treated endoscopically in three cases (in two cases by endoluminal vacuum therapy and in one using an over-the-scope clip) and surgically in two cases (one anastomosis redo and one discontinuity resection). One readmission in each case was due to constipation and diarrhea, respectively. The 30- and 90-day mortality rates were 0.5% (n = 1) and 1.3% (n = 3), respectively (Table 4).

**Table 4:** Postoperative outcomes

	Benchmark patients (n = 226)	Excluded patients (n = 96)	p value
Complications, n (%)			
Any type	66 (29.2)	37(38.5)	n.s
Minor (CDC Grades I–II)	34 (15.0)	13 (13.5)	n.s
Major (CDC Grades IIIA–IV)	32 (14.2)	24 (25.0)	0.066
Anastomotic leak	21 (9.3)	14 (19.7)	0.017
Urologic event	2 (0.9)	2(2.1)	
Pulmonary event	2 (0.9)		
Mechanical ileus	4 (2.8)	1 (1.4)	
Intraabdominal hematoma	2 (0.9)	1 (1.4)	
Wound dehiscence	2 (0.9)	1 (1.4)	
Stoma problems	2 (0.9)		
Intraabdominal infection	1 (0.5)	4 (4.2)	
Rectovaginal fistula	2 (0.9)		
Unspecified	27 (11.9)	2(2.1)	
Readmission rate within 90 days of discharge, n (%)	9 (4.0)	3 (3.1)	n.s
Related to rectum resection	7 (3.1)	3 (3.1)	
Unrelated to rectum resection	2 (0.9)	0	
Mortality, n (%)			
30-day	1 (0.5)	1 (1.4)	n.s
90-day	3 (1.3)	2(2.1)	n.s

*CDC* Clavien-Dindo classification, *n.s.* not significant

### Benchmark and excluded patients

The benchmark and comparison (n = 96) cohorts showed no statistical differences in age, BMI, gender, histologic entity, UICC stage, and tumor size (Table 2). In terms of ASA classification, the benchmark group was significantly healthier (*p* = 0.022) and less frequently pretreated with neoadjuvant therapy (30.5% vs. 51.0%, respectively; *p* = 0.001).

**Fig. 2:**
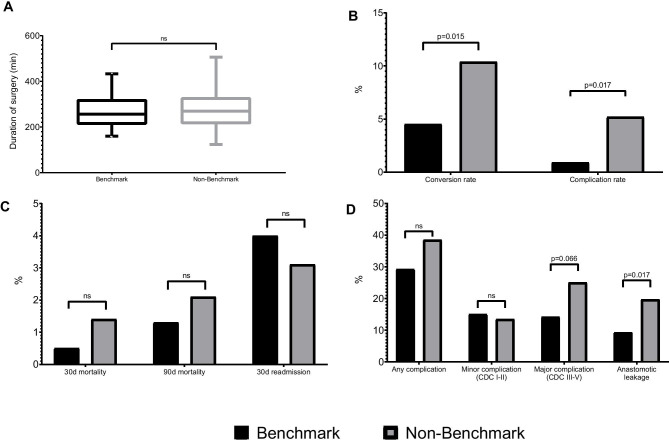
(**A**) Box plot graph with 10th to 90th percentile of duration of surgery in minutes. For clarity, statistical outliers were ignored. (**B**) Intraoperative conversion and complication rates (%). (**C**) 30- and 90-day mortality rate and 30-day readmission rate (%). (**D**) Postoperative complications divided into “any”, Clavien-Dindo classification I–II and III–IV, and anastomotic leakage rate. Ns, non significant

The inter-cohort operative time was similar between groups [266 (IQR 211–310) min in the benchmark and 276 (IQR 215–328) min in the comparison group]. However, in the benchmark cohort there was a significantly lower complication rate (0.9% vs. 5.2%, respectively; *p* = 0.017) and conversion rate (4.5% vs. 10.4%; *p* = 0.015) (Table 2). In terms of postoperative outcomes, the “any” complication rate was higher in the benchmark cohort but did not reach significance (38.5% vs. 29.2%, respectively; *p* = 0.066). However, the rate of insufficiency was more than twice as high in the comparison cohort compared with the benchmark group (19.7% vs. 9.3%; *p* = 0.017). This increased morbidity was not reflected in the rate of readmissions (4.0% vs. 3.1%, respectively) or in the 30- and 90-day mortality rates (0.5% vs. 1.4% and 1.3% vs. 2.1%, respectively) (Table 3 and Fig. 2).

### Oncological outcomes in benchmark and excluded patients

**Fig. 3:**
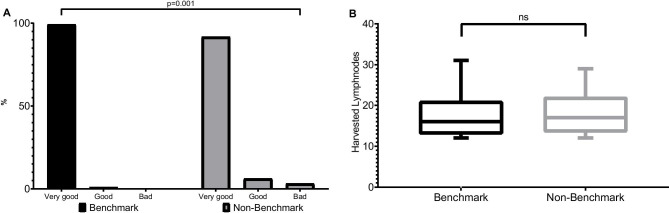
**A** Total mesorectal excision quality (%). **B** Box-plot graph of harvested lymph nodes with 10th to 90th percentile. Ns, not significant

There was no significant difference in terms of lymph node yield in the benchmark (19 (IQR 13–21)) and excluded (19 (IQR 14–22)) cohorts (Table 5). Although the R1 rate in the comparison group (3.1%) was more than three times higher than in the benchmark group (0.9%), the difference did not reach statistical significance. Very good TME quality was achieved in 99.1% of patients in the benchmark cohort (good TME quality in 0.9%) (Fig. 3). These results were significantly better than the TME quality in the comparison group (very good 90.6%, good 6.2%, poor 3.1%; *p* = 0.001). This is also reflected in the local recurrence rate, which was three times higher in the comparison cohort (5.6%) than in the benchmark group (1.5%) at a mean follow-up of 24.8 months (no significant difference). The overall survival, disease-free survival, and local recurrence rates were comparable between groups; however, there was a high rate of missing follow-up data in the benchmark (45.1%) and comparison (62.5%) groups.

**Table 5:** Oncological outcomes

	Benchmark patients (n = 226)	Excluded patients (n = 96)	p value
LN examined, median (IQR)	19 (13–21)	19 (14–22)	n.s
Positive resection margins, n (%)	2 (0.9)	3 (3.1)	n.s
TME quality, n (%)			0.001
Very good	224 (99.1)	87 (90.6)	
Good	2 (0.9)	6 (6.2)	
Bad	0	3 (3.1)	
Overall survival, n, (%)			n.s
1 year	113 (91.2)	34 (94.6)	
3 years	107 (86.1)	27 (75.6)	
Missing data, n (%)	102 (45.1)	60 (62.5)	
Disease-free survival, n (%)			n.s
1 year	120 (97.1)	36 (100)	
3 years	117 (94.6)	32 (89.9)	
Missing data, n (%)	102 (45.1)	60 (62.5)	
Local recurrence, n (%)	2 (1.6)	2 (5.6)	0.08

*IQR* interquartile range, *TME* total mesorectal excision

### Benchmark values

The 30-day benchmark values are based on the results of 226 patients from five centers (Table 6). The cutoff values for conversion and intraoperative complication rates were ≤ 4.0% and ≤ 1.4%, respectively. In terms of postoperative complications, the cutoff was ≤ 28% for “any” and ≤ 18.0% for major complications. The R0 and complete TME benchmark rates at 30 days were 100%, with a lymph node yield > 18. The benchmark for rate of anastomotic insufficiency was < 12.5% and 90-day mortality was 0%. Readmission rates should not exceed 4%.

**Table 6:** Benchmark results

Benchmark parameters	Benchmark values
Conversion rate	≤ 4.0%
Intraoperative complications	≤ 1.4%
R0 rate	100.0%
Complete TME	100.0%
Lymph node yield	≥ 18
Anastomotic leak	≤ 12.5%
Complications of any severity	≤ 28.0%
Major complications (CDC ≥ III)	≤ 18.0%
30-day mortality	0.0%
Hospital readmission	≤ 4.0%

Benchmark values are the 75th percentile of the median proportions, apart from lymph node yield which is the 25th percentile of the median proportion (the higher the number of lymph nodes yielded, the better)

*CDC* Clavien-Dindo classification, *TME* total mesorectal excision

## Discussion

Robotic-assisted rectal resection can achieve outstanding results when performed by an experienced surgeon at an expert center. To evaluate a newer procedure, evidence of “non-inferiority” compared to the gold standard is first needed. In a second step, superiority should be demonstrated in studies so that the newer intervention can be established as the gold standard after widespread standardization. This is exemplified by robot-assisted prostatectomy. Unfortunately, this concept of evaluation has some pitfalls. If complication rates are already low, a very large cohort is required to be able to prove a significant difference. In addition, the participating surgeons in a multicenter prospective comparative study would have to be experts in the new and old surgical procedures. This is hardly feasible with today’s standardized procedures and the specialization of hospitals and surgeons. Thus, another tool is needed to evaluate interventions.

Our study aimed to make this evaluation possible. It provides benchmark values for several clinically relevant endpoints that can be immediately adopted by other institutions. Our study corresponds in large parts to the proposal for a standardized benchmarking report, which was established in the context of major liver resections [7]. The strength of our study is that the patients were all operated according to a standardized surgical procedure by designated robotic experts in high-volume centers and the data were interrogated in a standardized manner. This allows first publication of the best achievable outcomes in robotic-assisted low anterior rectal resection.

In 2019, the results of the largest prospective, randomized multicenter study comparing RLAR with LLAR were published [5]. The endpoints analyzed were conversion rate (RLAR 12.2%, LLAR 8.1%), intraoperative (RLAR 14.8%, LLAR 15.3%) and postoperative (RLAR 31.7%, LLAR 33.1%) complication rates, as well as TME quality (very good: RLAR 76.4%, LLAR 77.6%). Notably, the conversion rate is associated with an increased rate of local recurrence, as well as increased morbidity and mortality [8–10]. All these results were inferior to our benchmark values, which demonstrate the advantage of the proven surgical robotic expertise in our centers. A limitation of this comparison is that rectal amputations were included in the ROLLAR RCT and surgeons at different stages of the learning curve participated in this RCT.

Compared to the meta-analysis by Han et al. (eight RCTs, 999 patients: RLAR 495, LLAR 504) [4], our median operative times were significantly longer (266 (IQR 211–310) min) than in the meta-analysis (211 (IQR 191–259) min), but with significantly lower rates of incomplete TME quality (benchmark cohort 0% vs. RLAR 22.2% and LLAR 25.65%) and a higher average lymph node yield (benchmark cohort 18 vs. RLAR 17.5 and LLAR 17).

In 2020, Diers et al. published their paper reporting the nationwide in**-**hospital mortality rate following rectal resection for rectal cancer [11]. They found a mortality rate of 1.5% in very high output centers (case load > 50 per year) and 1.4% in high output center (case load around 32 patients per year), but with approximately 15% of the cases being emergency procedures. The anastomotic leakage rate was 11.8% in the very high and 12.4% in the high output centers. Those results are similar to our benchmark values, but are hardly comparable because there was no differentiation in those leakage rates towards an open or laparoscopic approach and the performed resection (i.e., low anterior, anterior, tubular/segmental, or sigmoid/left resection). There are limitations to our study. Our data are from only one continent, whereas three are recommended [7]. There were also differences in the number of patients per center, with > 100 patients from one center, > 50 from two centers, and ≤ 30 from the last two. While this fact better reflects reality than results from a high-output center, some differences in terms of experience with the procedure must also be considered; there may also have been an influence of learning curves on our results. Furthermore, this inhomogeneity in numbers per center means that there is also increased case weighting. This is reflected in the intercentral comparison of the anastomotic leakage rate: two centers reported the same number of anastomotic leakages but with twice the number of patients in the center, and the insufficiency rate was twice as high in the smaller group. Another limitation is that we cannot exclude the possibility that complications may have been documented incorrectly or not at all, especially with regard to CDC grade I. From the benchmark proposal by Rössler et al., we know that there is often a lack of documentation of pathologically elevated laboratory values, for example [7]. This would mean that our complication rate of any severity would be falsely low. In addition, our benchmark cohort showed a low rate of neoadjuvant therapy. We could identify two possible explanations. The first is a potential understaging preoperatively. The second one could be upon patients’ request for a primary surgery. However, a further comparison between the clinical and pathological tumor stage should be performed. There was no selection for this, but it must be assumed that this resulted in a lower complication rate and higher lymph node yield (mean 19.5 in patients without neoadjuvant therapy vs. 17.9 in neoadjuvant-treated patients, without statistical significance). As a further weakness, the rate of oncological follow-up was unfortunately very low, so that only a weak statement can be made.

With increasing cost pressure for hospitals, clinics, and ultimately the individual surgeon, there is a need for publication of performance parameters. Performance measurements not only enable better argumentation regarding increased costs, but also allow patients to decide regarding the clinic, type of intervention, and ultimately their preferred surgeon, which significantly improves their autonomy [12].

## Conclusion

Our study is the first to provide benchmark values on the peri- and postoperative outcome of robotic-assisted rectal resection. Our benchmark cohort is based on databases of designated robotic experts from national expert centers. Critical patient selection, including no prior surgery, low comorbidity, and operated using a standardized technique, has allowed us to achieve “ideal” outcomes. However, the learning curve continues to be a factor that influences outcomes and only national centers could be recruited. Thus, it can be assumed that as national and international implementations of RLAR continue, and experience grows as a result, outcomes will also change, and this study will need to be updated. Nevertheless, we are convinced that these benchmark values will be used as comparison values for other centers and that the concept of benchmarking will continue to expand.

## Data Availability

The data are available on request.
